# A *Yersinia pestis tat* Mutant Is Attenuated in Bubonic and Small-Aerosol Pneumonic Challenge Models of Infection but Not As Attenuated by Intranasal Challenge

**DOI:** 10.1371/journal.pone.0104524

**Published:** 2014-08-07

**Authors:** Joel Bozue, Christopher K. Cote, Taylor Chance, Jeffrey Kugelman, Steven J. Kern, Todd K. Kijek, Amy Jenkins, Sherry Mou, Krishna Moody, David Fritz, Camenzind G. Robinson, Todd Bell, Patricia Worsham

**Affiliations:** 1 Bacteriology Division, The United States Army of Medical Research Institute of Infectious Diseases, Fort Detrick, Maryland, United States of America; 2 Pathology Division, The United States Army of Medical Research Institute of Infectious Diseases, Fort Detrick, Maryland, United States of America; 3 Center for Genome Sciences, The United States Army of Medical Research Institute of Infectious Diseases, Fort Detrick, Maryland, United States of America; 4 Office of Research Support, The United States Army of Medical Research Institute of Infectious Diseases, Fort Detrick, Maryland, United States of America; University of Helsinki, Finland

## Abstract

Bacterial proteins destined for the Tat pathway are folded before crossing the inner membrane and are typically identified by an N-terminal signal peptide containing a twin arginine motif. Translocation by the Tat pathway is dependent on the products of genes which encode proteins possessing the binding site of the signal peptide and mediating the actual translocation event. In the fully virulent CO92 strain of *Yersinia pestis*, the *tatA* gene was deleted. The mutant was assayed for loss of virulence through various *in vitro* and *in vivo* assays. Deletion of the *tatA* gene resulted in several consequences for the mutant as compared to wild-type. Cell morphology of the mutant bacteria was altered and demonstrated a more elongated form. In addition, while cultures of the mutant strain were able to produce a biofilm, we observed a loss of adhesion of the mutant biofilm structure compared to the biofilm produced by the wild-type strain. Immuno-electron microscopy revealed a partial disruption of the F1 antigen on the surface of the mutant. The virulence of the Δ*tatA* mutant was assessed in various murine models of plague. The mutant was severely attenuated in the bubonic model with full virulence restored by complementation with the native gene. After small-particle aerosol challenge in a pneumonic model of infection, the mutant was also shown to be attenuated. In contrast, when mice were challenged intranasally with the mutant, very little difference in the LD_50_ was observed between wild-type and mutant strains. However, an increased time-to-death and delay in bacterial dissemination was observed in mice infected with the Δ*tatA* mutant as compared to the parent strain. Collectively, these findings demonstrate an essential role for the Tat pathway in the virulence of *Y. pestis* in bubonic and small-aerosol pneumonic infection but less important role for intranasal challenge.

## Introduction

*Yersinia pestis* is the causative agent of plague and primarily a disease of rodents, transmitted typically by fleas. Clinical forms of the disease in humans are bubonic, septicemic, and pneumonic. Humans most often become infected by fleabites which manifests in the bubonic form of plague and is characterized by painful local lymphadenopathy, referred to as a bubo. Infection of the lymph node may disseminate throughout the body leading to septicemic plague. *Y. pestis* may also reach the lungs and lead to the pneumonic form of plague which could be spread human-to-human by the respiratory route. Pneumonic plague is the most severe and frequently fatal form of the disease [1], [2].

Similar to other bacteria, *Yersinia* species have several mechanisms for the transport and secretion of proteins. The most studied of these mechanisms is the type III secretion system which delivers *Yersinia* outer proteins (Yops) into host cells [3]–[5]. The general method bacteria, including *Yersinia*, use for secretion is the Sec system which transports proteins across the cytoplasmic membrane in an unfolded state [6]. In contrast to the Sec system, twin arginine translocation (Tat) system delivers folded proteins into the periplasm, using the proton motive force at the membrane [7]. Proteins to be translocated by the Tat system typically have a consensus signal sequence containing twin arginines at the N-terminal signal peptide. The genes encoding the Tat system include *tatA, tatB, tatC,* and *tatD* which may or may not be associated in an operon, depending on the bacterium. The TatB and TatC proteins possess the primary binding site of the signal peptide of the protein directed for translocation [8]–[10]; whereas TatA mediates the actual translocation event [11], [12]. Recent evidence also suggests that TatE may also play some role in the transport of Tat substrates [13]. Interestingly, the TatD protein is located within the cytoplasm and possesses DNase activity but is not required for Tat translocation of proteins [14].

The Tat system has been shown to be important for virulence in both animal and plant pathogens [15]–[25], including *Yersinia pseudotuberculosis* [26]. Therefore, the Tat system represents a potential therapeutic target for a wide range of bacterial pathogens. To determine what role this translocation system may play in virulence in *Y. pestis*, we deleted the *tatA* gene from the chromosome of the fully virulent CO92 strain [27] and found several dramatic phenotypic effects.

## Materials and Methods

### Bacterial strains and medium

The *Escherichia coli* and *Y. pestis* strains and plasmids used in this study are listed in Table 1. All *E. coli* strains were grown at 37°C on Luria-Bertani (LB) plates or in LB broth, and when needed, ampicillin was included at 50 µg/mL. For routine growth, the CO92 strain of *Y. pestis* was maintained on sheep blood agar plates or in heart infusion (HI) broth at 28°C. When grown at 37°C, medium was supplemented with either 2.5 mM CaCl_2_ or 20 mM MgCl_2_ and 20 mM sodium oxalate (MOX), as indicated. Selection of *Y. pestis* cointegrants or mutant strains occurred on LB Lennox agar (L2897; Sigma-Aldrich; Saint Louis, MO) plates supplemented with ampicillin (50 µg/mL) or 5% sucrose, as needed. To verify that the mutant retained the *pgm* locus, *Y. pestis* strains were screened on Congo Red agar plates [28]. To confirm that the bacteria isolated following animal challenges was *Y. pestis*, bacteria were screened on *Yersinia* selective agar plates which contains cefsulodin, irgasan, and novobiocin (R01988; Remel, Inc; Lenexa, KS).

**Table 1:** Strains and Plasmids.

	Relevant Characteristics	Reference/Source
*E. coli* strains		
DH5α		NEB
TransforMax EC100D	*pir*-116	EPICENTRE
*Y. pestis* CO92 strains		
wild-type	fully virulent	[27]
Δ*tatA*	*tatA* in-frame deletion mutant	this study
Δ*tatA*/pJET1.2+*tatA*	complemented Δ*tatA* mutant	this study
CO92-C12	F1 antigen negative strain	[57]
CO92 pLcr^-^	pLcr negative strain	USAMRIID collection
Plasmid		
pJET1.2	PCR cloning vector	Fermentas, Inc
pJET1.2+*tatA*	contains DNA fragment of *tatA* gene and flanking sequence	this study
pJET1.2+ Δ*tatA*	deletion of *tatA* from DNA fragment	this study
pWSK30	low copy *E. coli* vector for cloning	[29]
pWSK30+ Δ*tatA*	deletion of *tatA* from DNA fragment	this study
pCDV422	suicide vector containing Amp^R^ and *sacB*	[30]
pCDV422+Δ*tatA*	vector to introduce in-frame deletion of *tatA* from DNA fragment	this study

USAMRIID is compliant with all federal and Department of Defense regulations pertaining to the use of Select Agents.

### Growth assays

Growth assays were performed in HI broth at 28°C or 37°C with either CaCl_2_ or MOX, as indicated. Assays were performed using an Infinite M200 pro (Tecan; Männedorf, Switzerland) microplate reader in 96-well microtiter plates. The OD_600_ was measured every 60 min. For all assays, the bacterial strains were grown overnight at 28°C in HI broth and resuspended in fresh HI broth (alone or with CaCl_2_ or MOX as indicated) at an OD_600_ of ∼0.1. The resuspended cultures were diluted 1∶1 into the respective HI broth. For the assays performed at 37°C, the cultures were initially incubated at 28°C for 1 h for strain acclimation and then increased to 37°C. All samples were performed in quadruplicate and included medium controls to confirm sterility and for use as blanks to calculate the absorbance of the cultures. Additional well samples were included to monitor the increase of CFUs over the course of the growth study.

### Mutant construction

A fragment of DNA containing the *tatA* gene was PCR amplified (Table 2) using Phusion Taq polymerase (New England Biolabs, Inc.; Ipswich, MA) from genomic DNA from the CO92 strain of *Y. pestis*. The PCR product was ligated into pJET1.2 (Fermentas, Inc.; Glen Burnie, Maryland). The *tatA* gene was removed from the plasmid in-frame through inverse PCR (Table 2) while retaining the first and last three nucleotides of the gene. The *Y. pestis* DNA containing the deleted *tatA* gene was excised as an EcoRI fragment and cloned into pWSK30 [29]. The insert was then sub-cloned as a SacI-SalI fragment into vector pCDV422 [30].

**Table 2:** Primers.

Cloning *tatA*	
tatA 5′:	CCGCTCGAGACGCTGTCCCTGCCTCAAGTA
tatA 3′	CGGGATCCCGCGCCGTTTTAATATGGAAGTA
**Deleting ** ***tatA***	
tatA inverse 5′:	GCTGTGTTCGATATCGGGTTTA
tatA inverse 3′:	CATATTACCTACCTCTATTTATA
**Screening for mutant**	
tatA upstream:	TACACCCGCCCAACACAAGAGA
tatA downstream:	CGCGCCGTTTTAATATGGAAGTA
**Screening for pCD1**	
lcrV-1:	AGGGTGGAACAACTTACTG
lcrV-2:	GTGCCACTACTAGACAGATGC
**Screening for pMT**	
Ymt-5′	TTTCGGCCAATCTCCAACAGTA
Ymt-3′	TCCGACCGCCCACATCA
CapAG-5′	AAAAATCAGTTCCGTTATCG
CapAG-3′	CTGCCCGTAGCCAAGAC
**Screening for pPst**	
Pla-5′	TGGCTTCCGGGTCAGGTA
Pla-3′	AGCCGGATGTCTTCTCACG

Construction of the *Y. pestis* Δ*tatA* mutant was performed by the procedure as previously described [5], [31]. Briefly, the pCDV422+Δ*tatA* plasmid was introduced by electroporation into electrocompetent *Y. pestis* [32]. Cointegrates were selected on LB agar plates containing ampicillin. The cointegrate strain was grown overnight in HI broth and plated on LB agar plates containing 5% sucrose to select for allelic exchange recombinants. Deletion mutants of Δ*tatA* were identified by PCR (Table 2). The presence of the *Y. pestis* virulence plasmids was confirmed via PCR (Table 2). The Δ*tatA* mutant strain was next passaged through Swiss Webster mice by subcutaneous challenge, as routinely practiced by many labs [31], [33], [34], in order to ensure the genetic stability of the mutant strain after numerous *in vitro* growth steps needed to construct the mutant strain. The *Y. pestis* genome is unstable due to the presence of numerous IS*100* elements [35]–[37], and the passage of the mutant through an animal decreases the likelihood of a mixed population [2]. Animal passaged isolates of the Δ*tatA* mutant strain were harvested from the spleens of moribund mice following euthanasia.

To demonstrate phenotypes observed for the Δ*tatA* mutant were due specifically to the deletion of the gene, the mutant strain was complemented with an intact functional *tatA* gene present on pJET1.2 *in trans* via electroporation as described above.

### Microscopy

#### Light microscopy

*Y. pestis* strains were grown as indicated and fixed in 4% formaldehyde. The samples were then stained with Wayson stain [38] and observed with an Eclipse E800 fluorescence microscope (Nikon, Inc.; Melville, NY). Images were captured using a Microfire camera and Pictureframe software (Optronics; Goleta, California). Fluorescence microscopy. *Y. pesits* was grown at 37°C in HI broth in the presence of CaCl_2_ and fixed as described above. The presence of F1 antigen on *Y. pestis* was determined by indirect fluorescence microscopy (IFM) using the mouse monoclonal antibody (mAb) F1-04-A-G1 [39]. Samples were spun onto slides using a Cyto-Spin centrifuge (Thermo Shandon; Pittsburgh, PA), blocked with 7% skim milk in PBS for 30 min, exposed to the primary antibody (1∶5,000) in the presence of skim milk in PBS containing 0.1% Tween-20 (PBST) for 1 h, washed three times with PBST, incubated with a secondary antibody (goat-anti mouse antibody conjugated with Texas Red) in the presence of 7% skim milk in PBST for 1 h, washed again, and then observed as described above.

#### Electron microscopy

Standard methods for transmission electron microscopy (TEM) were employed. *Y. pestis* samples were fixed with 1% glutaraldehyde and 4% formaldehyde in 0.1 M phosphate buffer for several days and sterility was confirmed. Post fixation was performed for 1 h at room temperature in phosphate buffer containing 1% osmium tetroxide and contrasted in ethanolic uranyl acetate before dehydration in a graded series of ethanol rinses and propylene oxide. The samples were embedded into EMbed-812 embedding medium (Electron Microscopy Sciences; Hatfield, PA) overnight at room temperature and the samples were then sectioned into 90-nm sections. These sections were counterstained with uranyl and lead salts. For immuno-EM (IEM) analysis, samples were fixed in 0.1% glutaraldehyde and 4% paraformaldehyde in 0.1 M phosphate buffer for several days and sterility confirmed. Samples were dehydrated using graded ethanol, embedded in LR White resin (Polysciences Inc., Warrington, PA), and heat cured. Sections were cut as described above, blocked, exposed to primary antibody to F1 at a 1∶100 dilution, washed, and then incubated with the secondary antibody conjugated with 10 nm gold particles. Sections were counterstained with uranyl and lead salts. All EM samples were examined using a JEOL 1011 transmission electron microscope.

### Biofilm assay

*Y. pestis* strains were tested for biofilm formation and adherence. The methods followed were previously described [40], except that bacteria were grown in HI broth for 24 h and then diluted in 40% HI broth for biofilm measurements in 24-well polystyrene dishes. The bacteria were grown at 200 rpm for approximately 18 h at 26°C. The biofilm was stained with 0.01% crystal violet. The wells were washed three times and bound dye was solubilized with 80% ethanol-20% acetone. The crystal violet was assayed by absorbance. The standard deviation was derived from quadruplicate samples. These data represent three separate experiments.

### Protein extraction and analysis

Both whole-cell extracts and supernatants were collected for protein analysis from *Y. pestis* strains grown in HI broth at 28°C or at 37°C (with CaCl_2_ or MOX, as indicated) as previously described [5]. Briefly, whole-cell extracts from *Y. pestis* were obtained from collecting bacteria from 18 h grown cultures. The cultures were centrifuged, the supernatant fluids collected, and the pellets resuspended in 1 ml of ice-cold water. The cultures were pelleted, suspended in water with MPBio Lysing Matrix B (MP Biomedicals; Solon, OH), bead beat for 40 s with a FastPrep FP120 Cell Disrupter (MP Biomedicals), chilled on ice, bead beat again for 40 s, microfuged for 5 min, and then passed through a 0.2 micron filter. The preparation was then sterility checked by plating a portion of the sample on sheep blood agar plates. The *Y. pestis* supernatants were concentrated by passage through a centrifugal filter device (Amicon Ultra-10K, Millipore; Billerica, MA), heat fixed (95°C for 30 min), and sterility checked. The protein concentrations from all samples were determined using the BCA Protein Assay kit (Pierce; Rockford, IL) per the manufacture’s recommendations. Equal protein concentrations of samples were run on 10% Bis-Tris gels (Invitrogen; Carsbland, CA) and stained using the Gel Code Blue kit (Pierce).

For Western analysis, fractionated proteins were transferred onto a PVDF membrane overnight at 4°C in 1× NuPAGE transfer buffer (Invitrogen), 0.35% SDS, and 20% methanol. After transfer, the membranes were blocked with 10% skim milk in PBS containing 0.1% Tween-20. Mouse monoclonal antibodies [anti-F1, anti-GroEL (Enzo-Life Sciences, Plymouth Meeting, PA), or anti-LcrV [41]] were used at a dilution of 1∶5,000 and secondary rabbit anti-mouse horseradish peroxidase was used at a dilution of 1∶5,000. Bands were visualized using 4-chloronaphthol/3,3′-diaminobenzidine (Pierce). To demonstrate that the bands were the expected respective proteins, recombinant F1, GroEL, or LcrV were also run on the gels for control purposes.

### Animal challenges

To determine the LD_50_ values for the wild-type and Δ*tatA* mutant, at least 5 groups of 10 naïve female 6–8-week-old Swiss Webster mice were challenged by the various routes. For all methods of infection, the challenge doses were determined by serial dilutions and plating. Subcutaneous challenge (sub-Q). Frozen *Y. pestis* stocks were streaked onto tryptose blood agar base (Difco Laboratories, Detroit, MI) slants and incubated at 28°C for 2 days. Bacterial cells were harvested from the slants in 10 mM KPBS (potassium phosphate buffered solution), and mice were challenged with 0.2 ml aliquots at various cell concentrations. Aerosol challenge. For aerosol challenges, a suspension of *Y. pestis* prepared from a slant suspension was used to inoculate flasks containing 100 ml of HI broth at an approximate OD_600_ of 0.01. The broth cultures were grown for 24 h in a 30°C shaker at 100 rpm, centrifuged, washed twice with HI broth, adjusted for various challenge doses. Mice were exposed to *Y. pestis* using a dynamic 30-liter humidity-controlled Plexiglas whole-body exposure chamber, as previously described [42]. The calculated inhaled doses were obtained as previously described [42], [43]. Intranasal challenge. Mice were anesthetized with 150 µl of ketamine, acepromazine, and xylazine injected intramuscularly. The mice were then challenged by intranasal instillation with 50 µl of *Y. pestis* suspended in KPBS from slant grown cultures as described above. For all challenge experiments, mice were monitored several times each day and mortality rates (or euthanasia when moribund) were recorded. *In vivo* dissemination experiments. For *Y. pestis* dissemination studies after intranasal challenge, approximately equal numbers of either the parental (113,000 CFU) or Δ*tatA* mutant (76,000 CFU) strains were used to challenge mice as described above. At specified time points after challenge, mice (n = 4–5) were then humanely euthanized within a CO_2_ chamber. The lungs and spleens were harvested, rinsed with KPBS, weighed, and then homogenized in 1 ml of KPBS in a tissue grinder (Kendall Healthcare Precision Disposable Tissue Grinder Systems, Covidien; Mansfield, MA). The homogenates were then serially diluted and plated on to sheep blood agar plates.

Challenged mice were observed at least twice daily for 21 days for clinical signs of illness. Humane endpoints were used during all studies, and mice were humanely euthanized when moribund according to an endpoint score sheet. Animals were scored on a scale of 0–12∶0–3 = no clinical signs; 4–7 = clinical symptoms; increase monitoring; 8–12 = distress; euthanize. Those animals receiving a score of 8–12 were humanely euthanized by CO_2_ exposure using compressed CO_2_ gas followed by cervical dislocation. However, even with multiple checks per day, some animals died as a direct result of the infection.

Animal research at The United States Army of Medical Research Institute of Infectious Diseases was conducted and approved under an Institutional Animal Care and Use Committee in compliance with the Animal Welfare Act, PHS Policy, and other Federal statutes and regulations relating to animals and experiments involving animals. The facility where this research was conducted is accredited by the Association for Assessment and Accreditation of Laboratory Animal Care, International and adheres to principles stated in the Guide for the Care and Use of Laboratory Animals, National Research Council, 2011.

### Pathology

Postmortem tissues were collected from mice challenged with *Y. pestis*, fixed in 10% neutral buffered formalin, routinely processed, embedded in paraffin, and sectioned for hematoxylin and eosin (HE) staining as previously described [44]. Immunohistochemistry was performed on sections using a primary antibody to the F1 antigen and secondary antibody, a peroxidase-labeled polymer, EnVision Peroxidase kit (Dako Corp., Carpinteria, CA). The slides were stained with substrate-chromogen solution and counter stained with hematoxylin.

### Statistics

For comparing data from the biofilm experiments and TTD studies of mouse challenges, statistical significance (*P*<0.05) was determined by the two-tailed Student *t* test. LD_50_ analysis was determined by the Bayesian probit analysis. Survival rates were compared between groups by Fisher exact tests with permutation adjustment for multiple comparisons using SAS Version 8.2 (SAS Institute Inc., SAS OnlineDoc, Version 8, Cary, N.C. 2000).

### Screening the *Y. pestis* genome for Tat motifs

To scan for additional Tat-like motifs, we developed a script that allows more flexibility at each position in the motif with amino acids in the same basic grouping: non-polar, polar, acidic and basic. For positions with validated variants from multiple groups, we allowed substitution to either group. The script does not calculate total similarity distance from the original sequence allowing for multiple changes in order to give us the most flexibility possible within each grouping at each position. The resulting hits largely agree with the Hidden Markov Model results for the Tat signal motif available at Pfam (PF10518). Model data available at pfam.sanger.ac.uk/family/PF10518/hmm.

## Results

### Construction of the Δ*tatA* mutant

In *Y. pestis*, the *tatABCD* genes are clustered very closely together on the chromosome [45]. The *tatA* gene (267 bp) is 4 bp downstream of *tatB* (663 bps). Three bp downstream of *tatB* is the *tatC* gene (777 bp). The *tatD* gene is 540 bp in length and is 14 bp downstream of *tatC*. The *tatA* gene was deleted in-frame from the chromosome of the fully virulent CO92 strain of *Y. pestis*, as described above in the Materials and Methods section. Deletion mutants were screened by PCR using primers outside of the *tatA* cloning region (Table 2). For those clones which reverted back to the wild-type strain, a PCR fragment of 2.1 kb was observed. However, for those mutants deleted of the *tatA* gene, a shift of approximately 250 bp was observed (data not shown).

### Growth of the Δ*tatA* mutant in culture medium is not altered but cell morphology is

**Figure 1:**
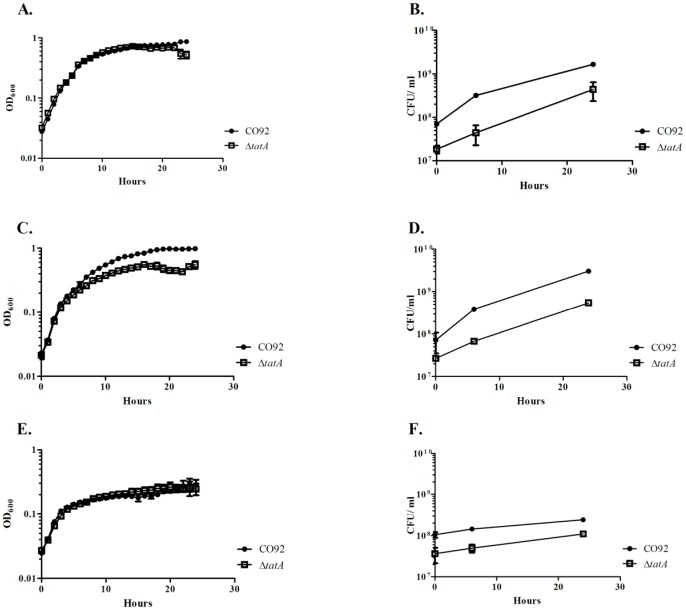
Growth assays. *Y. pestis* wild type and Δ*tatA* mutant strains were grown in HI broth at 28°C (A and B), 37°C with CaCl_2_ (C and D), or 37°C with MOX (E and F). Growth was monitored by both optical density (A, C, and E) and CFU counts (B, D, and F). OD measurements are based upon quadruplicate samples and bars represent standard deviation. CFU measurements are based upon triplicate samples and bars represent standard deviation. These data represent at least two separate experiments.

Growth curves were performed comparing the wild-type and Δ*tatA* mutant in HI broth (28°C, 37°C with CaCl_2_, and 37°C with MOX) by both optical density (OD_600_) and CFU counts (Fig. 1). No differences in growth rates were observed between strains as observed by OD (Fig. 1). However, CFU counts at all-time points (even for the starting inoculum at Time 0), were consistently lower in the mutant strain compared to the wild-type strain despite having similar OD measurements. However, the increase in CFU counts of both parent and mutant strains over the time course was still similar.

**Figure 2:**
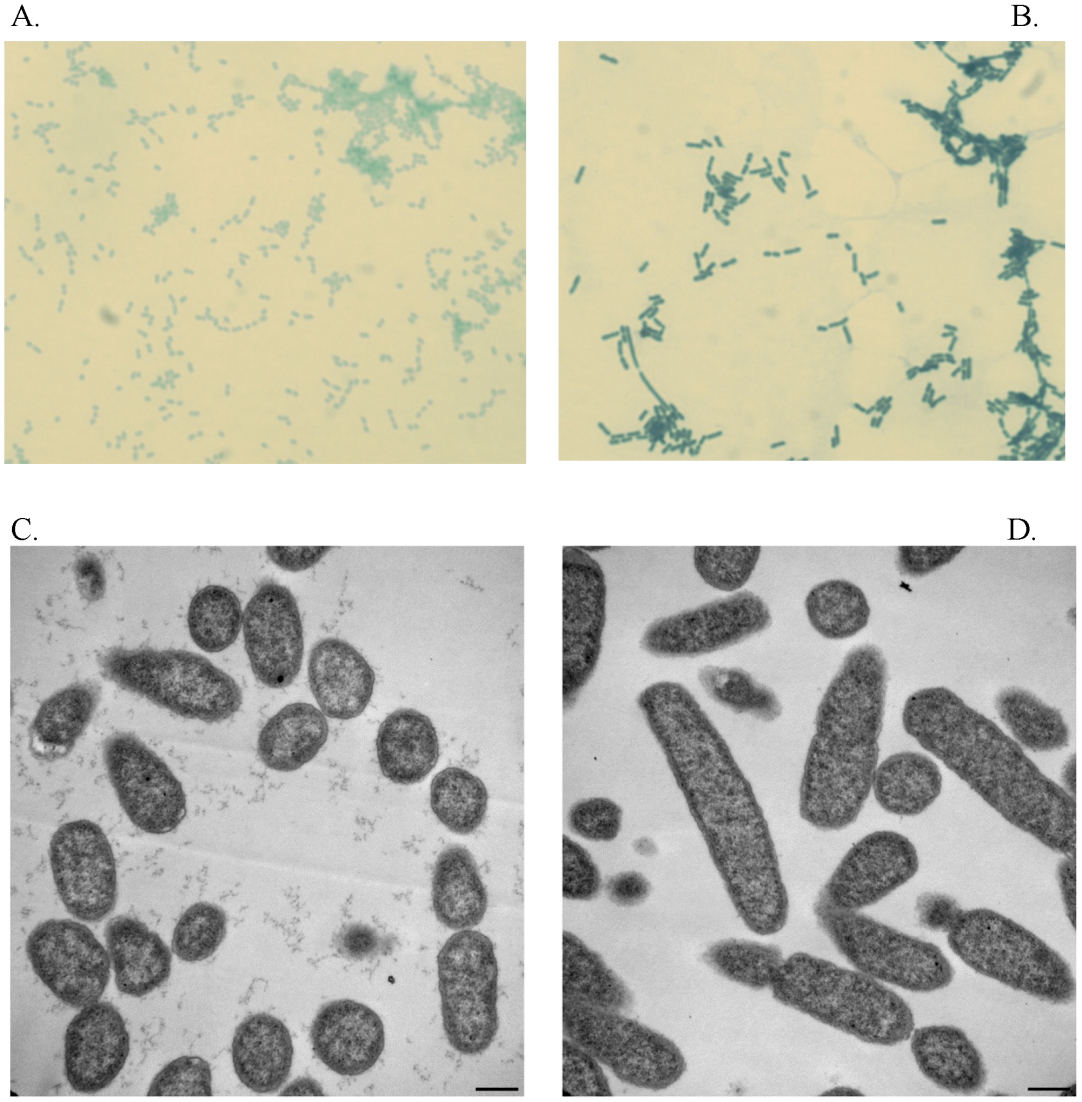
Morphology of *Y. pestis* strains. *Y. pestis* was grown at 37°C in presence CaCl_2_ and examined by microscopy. The samples, wild-type (A) and Δ*tatA* (B), were fixed, stained with Wayson stain, and examined by microscopy (100×). Additionally, samples, wild-type (C; micron bar = 0.5 µm) and Δ*tatA* (D; micron bar = 0.5 µm), were examined by TEM.

To determine why the CFU counts between the two strains were different; cells were examined by light microscopy. The mutant displayed an abnormal morphological phenotype at both 28°C (data not shown) and 37°C with CaCl_2_ (Fig. 2). The Δ*tatA* bacteria lost the typical *Yersinia* coccobacillus shape and became more elongated to a pronounced bacillus form, as compared to the wild-type strain (Fig. 2A and B). To further characterize the structural differences of the Δ*tatA* mutant strain, TEM was performed with the parental and mutant strains grown at 37°C with CaCl_2_ (Fig. 2C and D). Again for the Δ*tatA* mutant, the bacteria displayed an elongated bacillus form. In addition, many of the bacteria appeared to be inhibited in separating from one another during cellular division. This phenomenon is discussed further below; however, our morphological results are in general agreement with *tat* mutations in other bacteria [25], [46], [47].

### The integrity of biofilm in the Δ*tatA* mutant is affected

**Figure 3:**
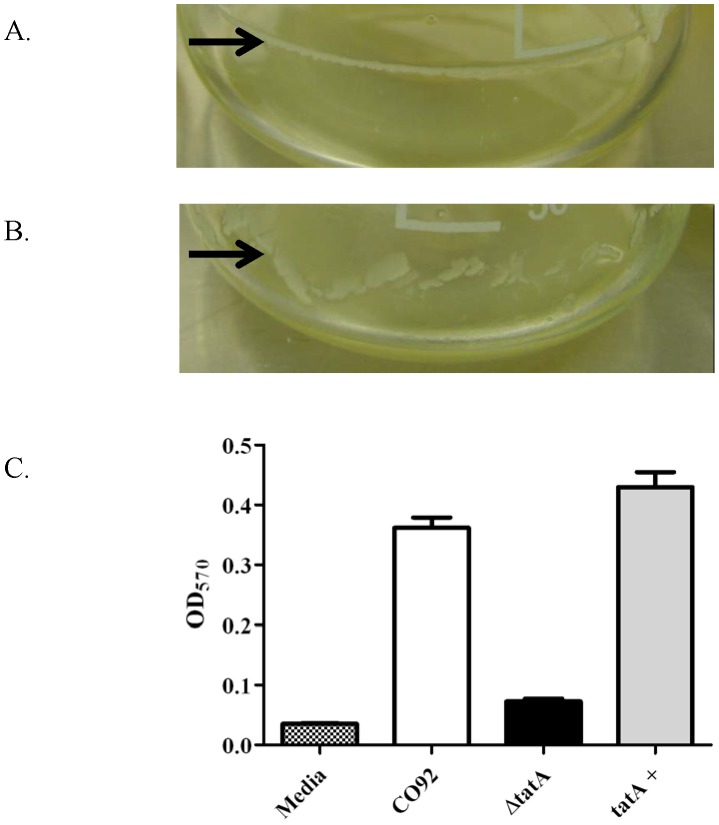
Adherence of *Y. pestis* biofilm. The *Y. pestis* A) wild-type CO92 and B) Δ*tatA* mutant cultures were grown in HI broth at 26°C. Arrows indicate biofilm formation. C) To measure biofilm adherence, the biofilm of wild-type CO92, Δ*tatA* mutant, and complemented Δ*tatA* mutant was stained with crystal violet and assayed by absorbance as described in the Materials and Methods. The standard deviation was derived from quadruplicate samples. These data represent three separate experiments.

In addition to the structural differences observed with the Δ*tatA* strain, the mutant also demonstrated a defect in biofilm integrity. After overnight growth at 26°C, a bacterial biofilm formed for both the *Y. pestis* wild-type (Fig. 3A) and mutant (Fig. 3B) cultures at the liquid/air interface. The biofilm, however, of the Δ*tatA* cultures was easily dislodged as compared to the wild-type strain.

To quantitate the adherence defect of the Δ*tatA* mutant, it was assayed as previously described [40]. As shown in Fig. 3C, the quantity of biofilm detected between wild-type *Y. pestis* and the Δ*tatA* mutant differed significantly (*P* = <0.0001). As described above, the Δ*tatA* bacteria were still able to produce biofilm; however, there was a defect in the adherence or integrity of the biofilm to bind to the substrate. In this assay, the biofilm was dislodged during the wash steps and prior to the absorbance readings. To demonstrate this adherence defect was due specifically to the deletion of *tatA*, the mutant was complemented with a functional *tatA* gene. As shown in Fig. 3C, the quantity of measured adherent biofilm for the complemented mutant strain and wild-type CO92 did not differ significantly (*P* = 0.09). Therefore, providing a functional *tatA* gene was able to restore the adherence/integrity defect of the Δ*tatA* mutant strain.

### The Δ*tatA* mutant is altered in F1 antigen localization

**Figure 4:**
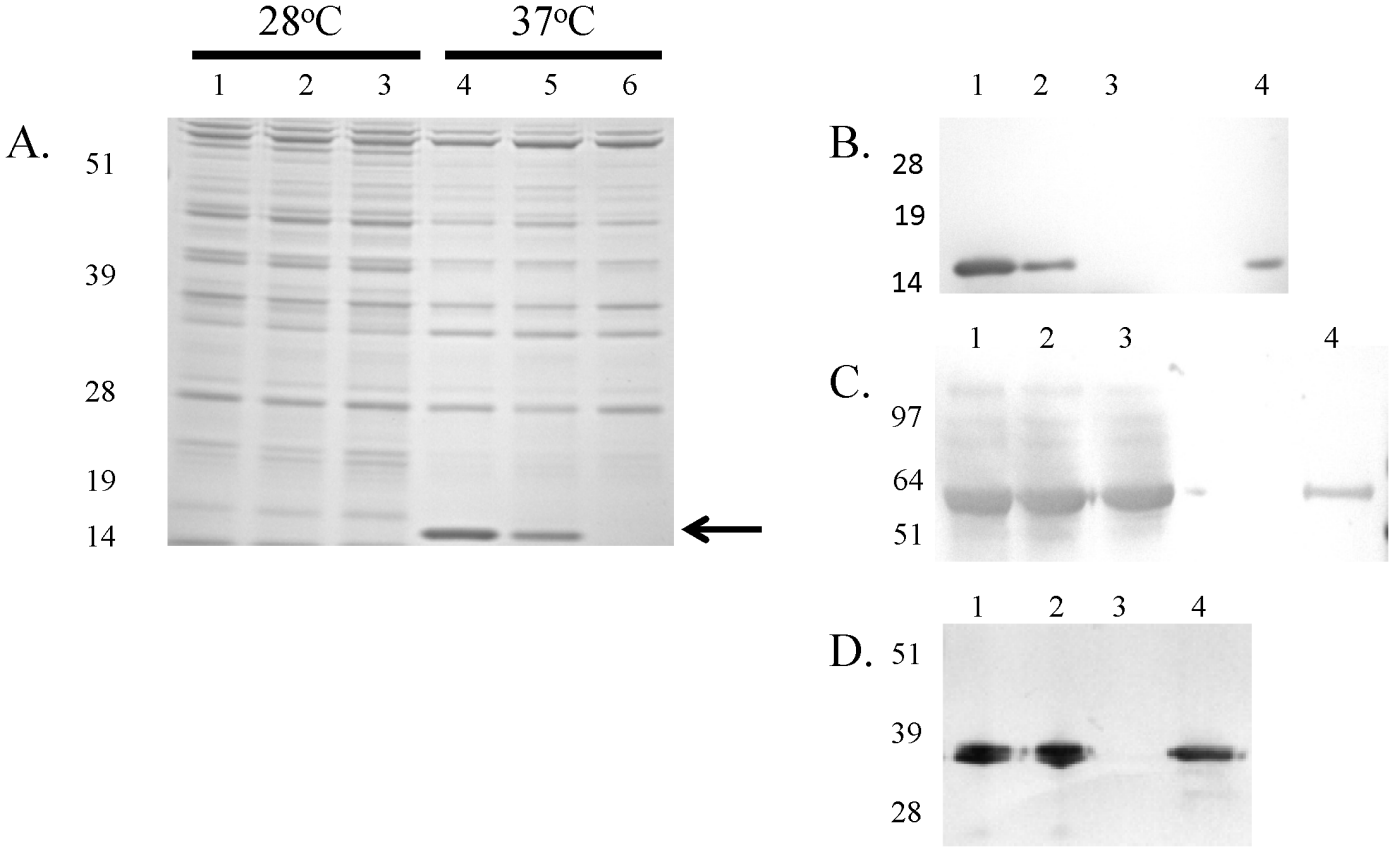
Analysis of *Y. pestis* proteins. A) Strains (wild-type, lanes 1 and 4; Δ*tatA*, lanes 2 and 5, and C12, lanes 3 and 6) were grown in HI broth at 28°C or 37°C containing CaCl_2_, as indicated. Proteins were extracted and ran on a SDS-PAGE gel at equal concentrations. The arrow indicates a protein band that is synthesized at 37°C for the CO92 and to a lesser extent the Δ*tatA* mutant but not in C12, a F1-antigen mutant strain. Molecular masses are indicated on the left in KDa. Strains were grown in HI broth at 37°C containing CaCl_2_ and proteins extracted for Western analysis. Equal concentrations of proteins from *Y. pestis* strains were blotted with a monoclonal antibody to either the F1 antigen (B) or GroEL (C). Lanes: 1, wild-type; 2, Δ*tatA*; 3, C12; and 4, recombinant protein (F1 antigen or GroEL, respectively). Molecular masses are indicated on the left in KDa. D) Strains were grown in HI broth at 37°C in the presence of MOX to create low calcium conditions. Equal concentrations of proteins from *Y. pestis* strains were blotted with an antibody to LcrV Lanes: 1, CO92 wild-type; 2, Δ*tatA*; 3, *Y. pestis* pLcr^-^; and 4, rLcrV protein. Molecular masses are indicated on the left in KDa.

As the deletion of the *tatA* gene would affect translocated proteins, we examined proteins extracted from both the cell pellet and supernatant fractions of mutant and wild-type bacteria grown in HI broth at 28°C or 37°C containing CaCl_2_. As shown in Fig. 4A, the proteins collected from pellets of *Y. pestis* grown at 28°C were separated on a 10% Bis-Tris gels, but no obvious differences were observed in the protein profile between wild-type, Δ*tatA*, or C12 (F1 antigen/*caf1* mutant) strains. However, when the protein pellets were examined from *Y. pestis* grown at 37°C under high calcium conditions, an intense band at <19 kDa appeared for wild-type CO92 which was not present for the 28°C samples. For proteins extracted for the Δ*tatA* pellet, this band was also present but not as intense as observed for the wild-type strain. We presumed that this band corresponded to the F1 antigen protein encoded by the *caf1* gene which is expressed by *Y. pestis* at 37°C and has a molecular mass of 15.5 kDa [48]–[50]. Further demonstrating that this band corresponded to the F1 antigen, it was absent from the protein profile of the C12 strain of *Y. pestis* (Fig. 4A).

Likewise, when pelleting *Y. pestis* cultures after growth at 37°C with calcium, wild-type *Y. pestis* formed a flocculent pellet (data not shown). In contrast, when the Δ*tatA* mutant was grown similarly, a very tight pellet was formed, similar to pelleted cultures of C12, the F1antigen mutant (data not shown). This defect was due specifically to the loss of the *tatA* gene since a complemented mutant would once again form a flocculent pellet similar to wild-type cultures (data not shown).

When performing Western blot analysis with a monoclonal antibody to the F1 antigen with equal amounts of proteins extracted from the pellets of CO92, Δ*tatA*, and C12 strains, similar results were obtained (Fig. 4B). A large prominent band was observed for the wild-type strain, a weaker band for the Δ*tatA* mutant, and no band was apparent for the F1 antigen mutant. Recombinant F1 antigen was also included as a positive control and corresponded in size to the bands observed for the bacterial pellets (Fig. 4B). Similar results were observed with the discrepancy between the amount of F1 antigen observed in the supernatants collected from wild-type and Δ*tatA* mutant cultures (data not shown). In addition, a control Western blot was performed using the same amount of protein but probing with a monoclonal antibody to GroEL, a 56-kDa molecular chaperone [51]. As shown in Fig. 4C and in contrast to the results observed with the anti-F1 antigen, no difference in the amount of GroEL was observed between strains.

To determine if synthesis or secretion of the V-antigen/LcrV, one of the *Yersinia* Yops which is surface exposed and serves many roles, one being necessary for translocation of the effector Yops [52]–[54], was also affected by deletion of the *tatA* gene; *Y. pestis* strains were grown at 37°C with MOX to induce expression of the low calcium response [4]. Proteins were collected from cell pellets, ran on SDS-PAGE gels, and tested by Western analysis using a monoclonal antibody to the V-antigen. As shown in Fig. 4D, no differences were observed for the presence of the V-antigen between strains in cell associated proteins, except for the *Y. pestis* strain which lacks the pLcr plasmid. Similar results were observed for proteins from culture supernatants (data not shown).

**Figure 5:**
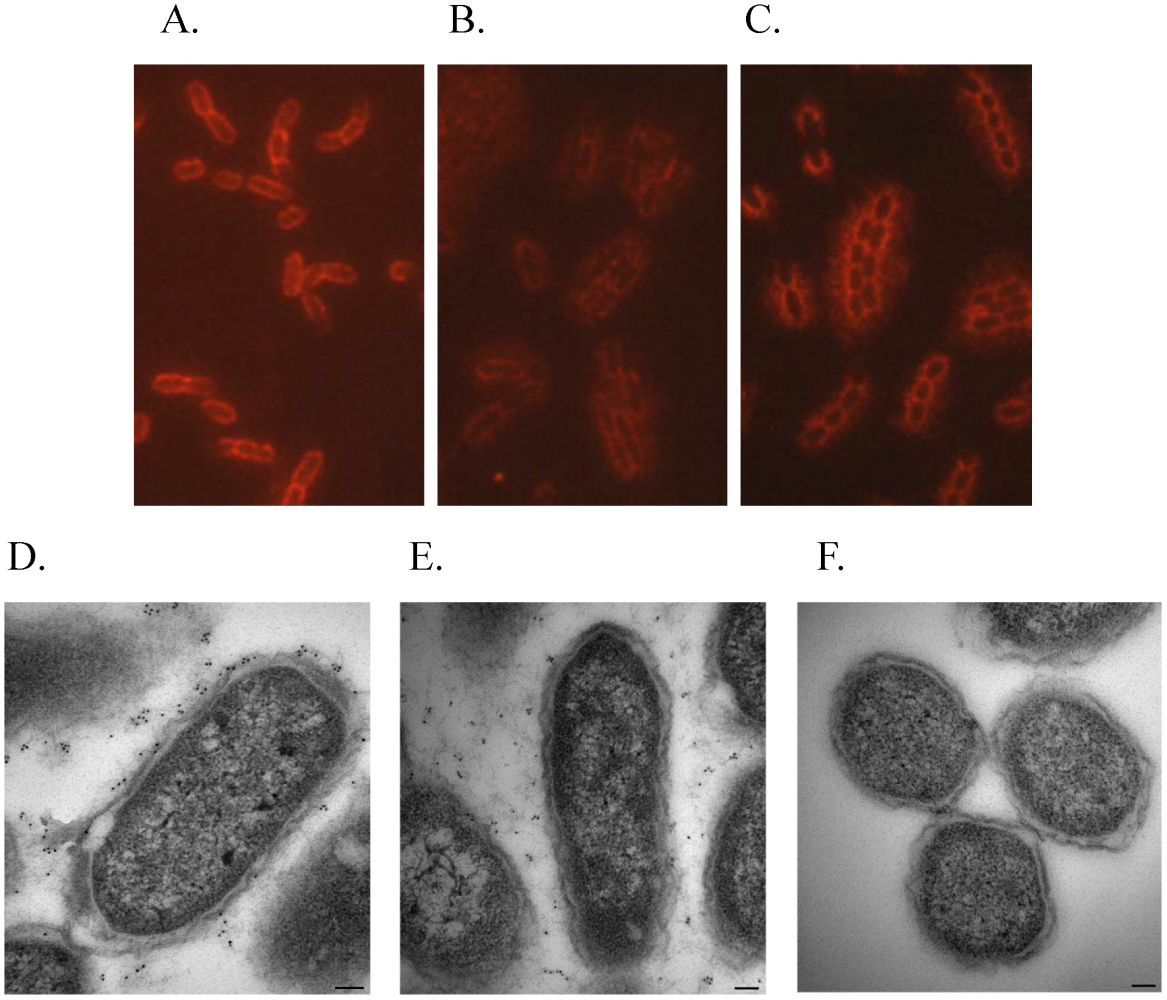
Localizing the F1 capsule by microscopy. *Y. pestis* strains were grown at 37°C in HI broth containing CaCl_2_ and exposed to an antibody against the F1 capsule. IFM samples: A) wild-type, B) Δ*tatA* mutant, and C) complemented Δ*tatA* mutant. The images (100×) were captured for all samples at identical camera settings to maintain relative fluorescence. IEM samples: D) wild-type (micron bar = 0.1 µm), E) Δ*tatA* mutant (micron bar = 0.1 µm), and F) C12, F1 negative (micron bar = 0.5 µm).

To attempt to define this defect in the concentration of the F1 antigen for the Δ*tatA* mutant, *Y. pestis* cells were examined by microscopy using both IFM and IEM. As shown in Fig. 5A, when wild-type CO92 was grown under conditions to promote F1 antigen synthesis and visualized by IFM using an antibody specific to the F1 antigen, the cells displayed a tight layer of fluorescence that surrounded the cells and in close association to the membrane. The Δ*tatA* mutant was grown under identical conditions but was only slightly fluorescent, indicating the presence of low but still detectable levels of F1 antigen. (Fig. 5B) However, fluorescence was not as intense as observed with the parental strain. In addition, the labeling for the mutant appeared to be more diffuse around the cells and not as tightly associated. To determine if this defect was due specifically to the deletion of the *tatA* gene, we observed the complemented mutant strain by IFM (Fig. 5C). As expected, the fluorescence of the complemented Δ*tatA* strain was restored to the intense fluorescent labeling that was associated with the wild-type bacterial cells. Also included in this study was the C12 strain which lacks the F1 antigen protein and no fluorescent labeling of this strain was observed (data not shown).

We further confirmed these studies by IEM. As shown in Fig. 5D, wild-type cells were decorated with gold bead labeling, indicating the presence of the F1 antigen, both in contact with the outer bacterial membrane and surrounding the outer area. In contrast, the gold labeling observed with the Δ*tatA* mutant was much less intense than the wild-type strain. The gold beads observed were rarely in association with the membrane but appeared diffuse away from the cell (Fig. 5E). The control for this study to demonstrate specificity of the labeling for the F1 antigen was the C12 strain. As shown in Fig. 5F, no gold beads were found associated with this strain.

### The Δ*tatA* mutant is attenuated in both bubonic and aerosol administered inhalational models of plague infection but not as attenuated following intranasal challenge

**Figure 6:**
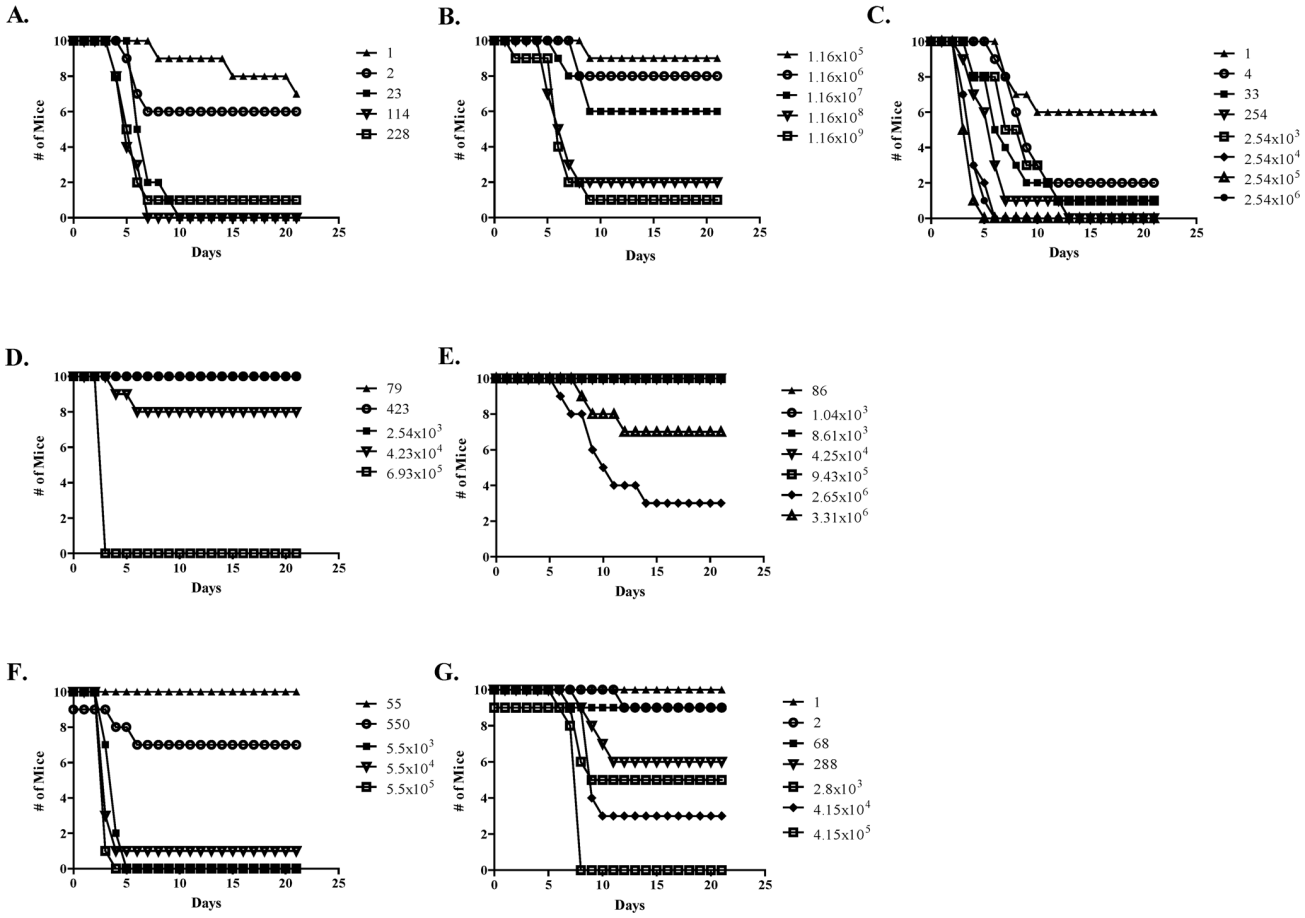
Animal challenge data. Groups of Swiss Webster mice were challenged and survival monitored following various plague models of infection (sub-Q injections A–C; small-particle aerosol D and E; and intranasal F and G) with various strains of *Y. pestis* (wild-type A, D, and F; Δ*tatA* mutant B, E, and G; and the complemented Δ*tatA* mutant C). The calculated LD_50_ values are included in Table 3 3.

The virulence of the Δ*tatA* mutant was assessed and compared to wild-type CO92 challenge through bubonic and pneumonic murine models (small-particle aerosol and intranasal instillation) of plague infection. From these assays, survival following challenge is shown in Fig. 6, and the LD_50_ values were determined for each route and summarized in Table 3.

**Table 3:** Calculated LD_50_ for the CO92 wild-type and Δ*tatA* mutant *Y. pestis* strains.

Strain	LD_50_ sub-Q.	LD_50_ aerosol^a^	LD_50_ intranasal
Wild-type CO92	∼2 CFU	6.7×10^4^ CFU	1.4×10^3^ CFU
Δ*tatA*	1.46×10^7^ CFU	>9.43×10^5^ CFU	2.4×10^3^ CFU
Δ*tatA* complement	∼1 CFU^b^	ND^c^	ND^c^

a Calculated inhaled dose.

b A significant difference was observed between the LD_50_ determinations between the Δ*tatA* mutant and complemented strain (*p*<0.0001).

c Not determined.

The LD_50_ for the CO92 wild-type strain by the bubonic challenge route with Swiss Webster mice was determined to be only ∼2 CFU (Fig. 6A and Table 3), in general agreement with previous estimates [55]. In contrast, the LD_50_ for Δ*tatA* mutant was calculated to be 1.46×10^7^ CFU (Fig. 6B and Table 3). To demonstrate this severe attenuation was due specifically to the deletion of the *tatA* gene, mice were then challenged with the complemented mutant. As expected, the LD_50_ for the complemented Δ*tatA* strain was restored to wild-type levels and determined to be ∼1 CFU (Fig. 6C and Table 3).

Next, mice were challenged by aerosol exposure with either the wild-type or Δ*tatA* to determine if the mutant was also attenuated by this model of pneumonic challenge. The LD_50_ for the CO92 strain was calculated to the 6.7×10^4^ CFU (Fig. 6D and Table 3), in general agreement with our previously published value of 6×10^4^ CFU [31]. In contrast, no mice succumbed to infection when challenged with the aerosolized Δ*tatA* at an estimated inhaled dose up to 9.43×10^5^ CFU. When mice were challenged at two additional higher doses (2.65×10^6^ and 3.31×10^6^), mortality was observed. However, the mice did not succumb to infection in a dose dependent manner (Fig. 6E). Therefore, we were not able to calculate a valid LD_50_ measurement. Regardless, the LD_50_ for the Δ*tatA* mutant would be 9.43×10^5^ CFU.

Surprisingly, when performing intranasal challenges as an additional method of pneumonic infection with the wild-type and mutant, little difference was observed between the calculated LD_50_ values between strains, 1.4×10^3^ CFU versus 2.4×10^3^ CFU, respectively (Figs. 6F,G and Table 3). Likewise, no differences in survival between groups of mice challenged at approximately equal doses between the two strains were noted except for the ∼10^3^ CFU challenge group (Table 4). In contrast, we observed a significant difference in TTD for mice challenged with the Δ*tatA* as compared to the wild-type strain (Table 4). Mice challenged intranasally with Δ*tatA* succumbed to infection at significantly later time points as compared to mice challenged with the wild-type strain at all doses (Table 4).

**Table 4:** Survival and TTD comparisons between mice challenged intranasally with the Δ*tatA* mutant and wild-type *Y. pestis* strains.

Strain	Dose (CFU)	Total	Alive	% Survival	p value	TTD±SD^a^	p value
CO92	550	9	7	78	0.63	5.00±1.14	0.0402*
*ΔtatA*	288	10	6	60		9.25±1.71	
CO92	5,500	10	0	0	0.01*	3.90±0.74	<0.0001*
*ΔtatA*	2,880	9	5	56		8.25±0.50	
CO92	55,000	10	1	10	0.582	3.22±0.44	<0.0001*
*ΔtatA*	41,500	10	3	30		8.86±0.90	
CO92	550,000	10	0	0	1	3.10±0.32	<0.0001*
*ΔtatA*	415,000	10	0	0		7.70±0.67	

a Time to death ± standard deviation in days.

*Statistical differences were considered significant when *p*<0.05.

The course of disease was very different for mice challenged intranasally with the Δ*tatA* mutant as compared to mice challenged with CO92. Whereas wild-type challenged mice succumbed to infection between 3–5 days (Table 4), approximately 1 week post challenge, the mice infected with the Δ*tatA* mutant began to display signs of inner ear infection: holding their head at a tilt and then progressing to whole body spinning. However within a day or two, some mice clinically improved, even though residual tissue damage was noted during histological evaluation (discussed below). Other mice eventually succumbed to infection or were euthanized when moribund.

**Figure 7:**
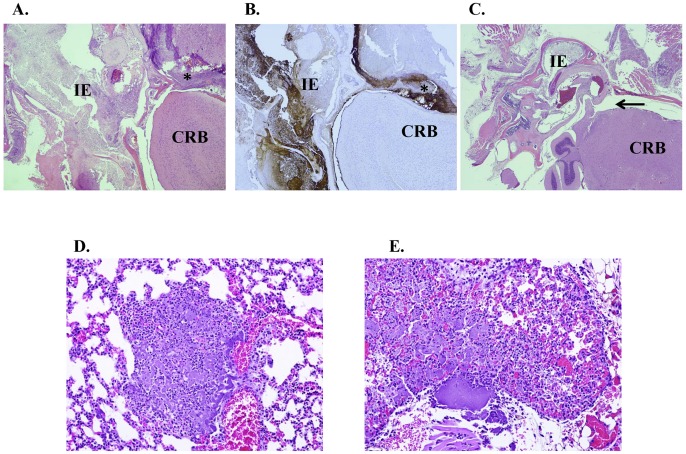
Pathology of mice challenged intranasally with *Y. pestis*. A and B) Skull sections (4×) of a mouse challenged with the Δ*tatA* mutant (2,800 CFU). The mouse was moribund and euthanized on day 8 postchallenge. Note inflammation (indicated by *) that extends into the cranial vault and meninges. Panel A shows HE staining, and Panel B shows IHC staining with antibody to the F1 capsule. C) HE stained section from a mouse (2×) challenged with wild-type *Y. pestis* (5,500 CFU) that succumbed to infection on day 3 post challenge. In contrast to panel A, the inflammation for mice challenged with the wild-type strain, is contained in the inner ear area and did not extend into the brain (arrow). D and E) Lung sections of mice challenged intranasally with *Y. pestis*. Overall, lung lesion development and character are the same between strains with necrosuppurative inflammation surrounding large colonies of bacteria. Panel D is of a lung section stained with HE (20×) from a mouse challenged with the Δ*tatA* mutant. Panel E is of a lung section stained with HE (20×) challenged with wild-type *Y. pestis*. IE = inner ear; CRB = cerebrum.

Histologic examination of cranial sections of mice infected intranasally with the Δ*tatA* mutant (Fig. 7A) or the wild-type CO92 strain (Fig. 7C) showed severe middle ear involvement, which was usually bilaterally present. The high incidence of infection/inflammation of the inner and external ear canal was most likely related to the intranasal route of exposure. Lesions were characterized by necrosuppurative inflammation with marked bacterial colonization in the associated bone marrow within the tympanic bulla, inner ear, and/or external ear (Fig. 7A–C). Bacteria were confirmed to be *Y. pestis* through immuno-histochemical (IHC) staining with antibody to the F1 capsule which is specific for *Y. pestis* [56], [57] (Fig. 7B).

Histologic examination of many of the mutant challenged mice (8/10) that succumbed to infection revealed moderate meningitis of the ventral meninges of the cerebrum and/or cerebellum. The areas of meningitis were characterized by necrotic debris and neutrophils admixed with fibrin, coccobacilli, and congested vessels (Fig. 7A and B). The meningitis in the ventral aspect of the cerebrum/cerebellum is probably an extension of inflammation/necrosis from the nasopharynx/nasal cavity and/or middle/inner ear through the osseous structures of the cranium. Again, the bacteria were confirmed to be *Y. pestis* through IHC staining with anti-F1 antibody (Fig. 7B). In addition, three of the mice which survived 21 days post challenge with higher doses of the Δ*tatA* mutant still retained a head tilt and histologic lesions consisted of meningitis characterized by necrosis and neutrophilic inflammation admixed with macrophages and fibrous connective tissue (data not shown).

In the case of CO92-infected mice, disease progression following intranasal instillation was much quicker as compared to challenge with the mutant strain, and the resulting illness was acute. In wild-type exposed mice, the presence of *Y. pestis* in the inner ear did not progress to meningitis because the time from exposure to the succumbing to infection was too short for lesions to develop (Fig. 7C), as opposed to what was observed with mice infected with the Δ*tatA* mutant strain (Fig. 7A). The lungs of the intranasal challenged mice from both the wild-type and the mutant were also examined. Overall, lung lesion development and characteristics were the same between mice challenged with either strain (Figs. 7 D–E).

**Figure 8:**
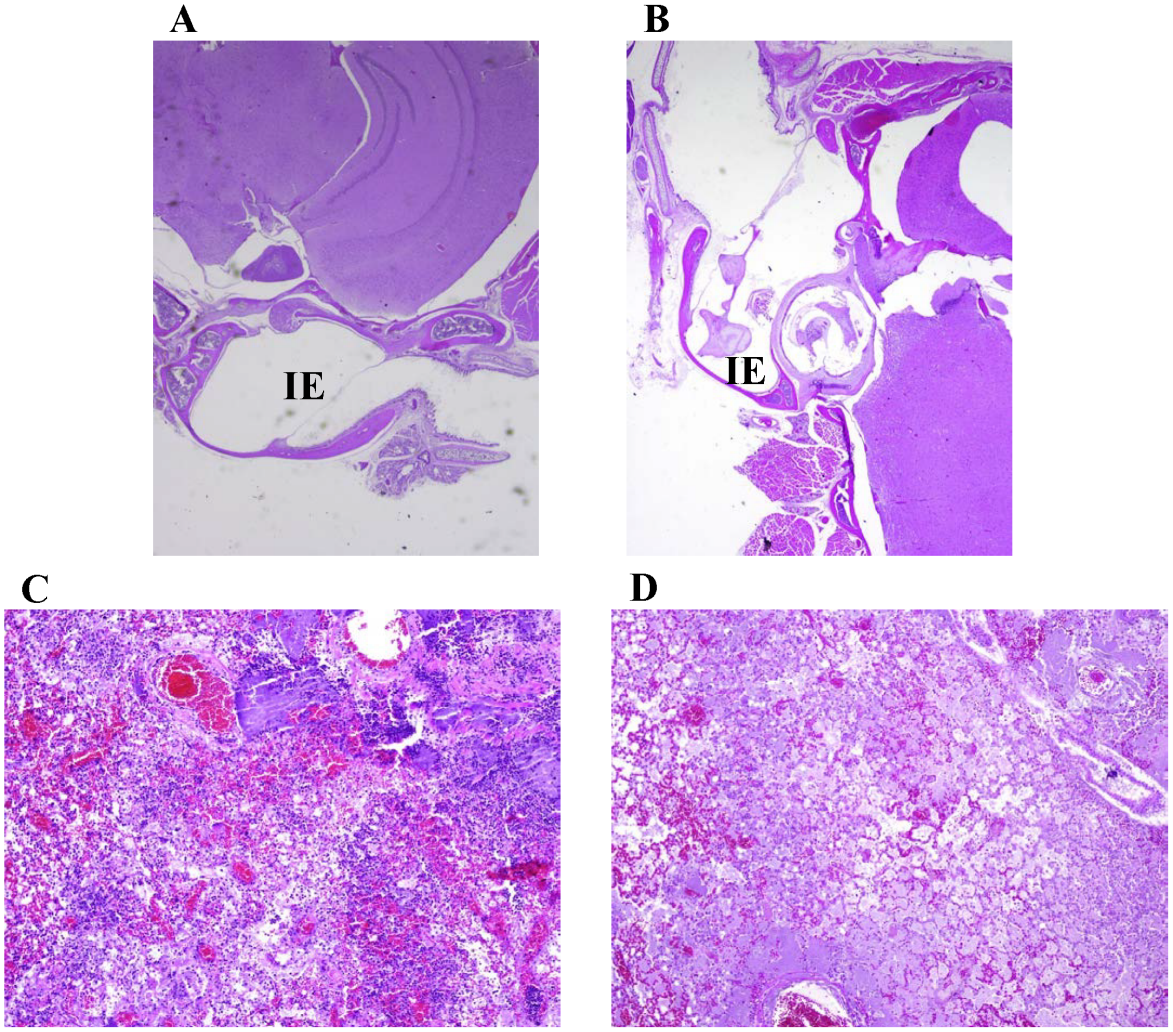
Pathology of mice challenged with *Y. pestis* by small particle aerosol. A and B) HE stained skull sections of aerosol challenged mice. A) Mouse (2×) challenged with wild-type CO92 (6.9×10^5^ CFU) that was moribund and euthanized on day 3 postchallenge. B) Mouse (4×) challenged with Δ*tatA* mutant (3.3×10^6^ CFU) that was moribund and euthanized on day 7 postchallenge. No inner ear (IE) or meningeal involvement was detected for mice aerosol challenged with either strain of *Y. pestis*. HE stained lung sections (10×) of mice aerosol challenged with CO92 (C) or Δ*tatA* mutant (D). There was no difference in lesion character or severity in mice challenged with either strain.

To determine if the presence of *Y. pestis* bacteria within the inner ear was specific to exposure by the intranasal route, pathology samples were also examined from mice challenged by small particle aerosol with either the wild-type or mutant strain. In contrast to the observations with intranasal challenged mice (Fig. 7A–C), no inflammation or bacterial colonization was observed in the inner ear or meningeal areas for mice challenged via small-particle aerosol delivery for the *Y. pestis* strains (Figs. 8A and B). Additionally, lungs were examined from mice challenged by aerosol with each strain (Figs. 8C and D). Again, there was no difference in lung lesion characters or severity in mice challenged between strains.

### The recovery and dissemination of the Δ*tatA* mutant is hindered after intranasal challenge

**Figure 9:**
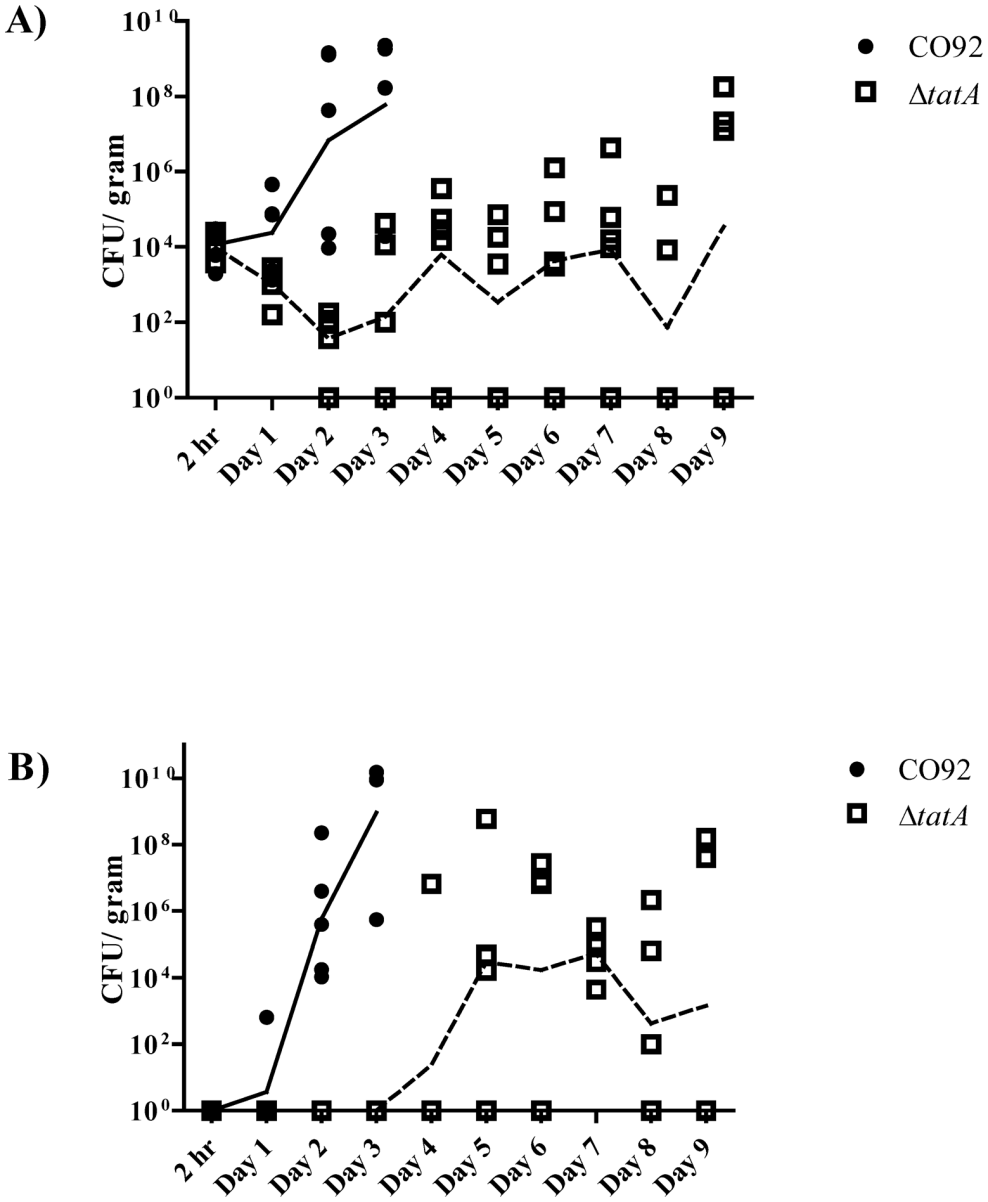
Dissemination studies of mice challenged intranasally with *Y. pestis*. Mice were challenged with CO92 (113,000 CFU) or Δ*tatA* mutant (76,000 CFU). At set time points, mice were euthanized, and the lungs and spleens were harvested. The A) lungs and B) spleens were homogenized and plated to determine bacterial recovery. For each time point, five mice were assayed, except for day 3 for wild-type challenged mice. Only four mice were tested as the remaining challenged mice had succumbed to infection. The lines (solid = CO92 and hashed = Δ*tatA*) are connecting at the geometrical means at the data points of CFU recovery from the respective organs are represent the overall trend during the course of infection.

Since there was little difference in the intranasal LD_50_ calculated between strains but a significant increase in TTD for mutant challenged mice, the dissemination of wild-type and mutant bacteria following intranasal challenge was compared. Groups of mice were separately challenged with approximately equal doses of either CO92 (113,000 CFU) or the Δ*tatA* mutant (76,000 CFU). For mice challenged with wild-type CO92, CFU recovery from the lungs showed some progression by day 1 and then increased rapidly for days 3 and 4 for all mice tested (Fig. 9A). By day 3, only four mice were alive for testing, while the remaining animals had succumbed to infection. For recovery of CFU from the spleen, wild-type bacteria were detected for one mouse by day 1; however, spleens for all mice had increased bacterial burden loads by days 2 and 3 (Fig. 9B).

In contrast, mice challenged intranasally with the Δ*tatA* mutant were severely delayed in progression of bacterial dissemination. Recovery of bacteria from the lungs 2 h postchallenge did not differ greatly between the wild-type and mutant strains (5.8% versus 4.2%, respectively) (Fig. 9A). Therefore, differences in tissue tropism or bacterial adherence to host cells after initial exposure would not appear to be a factor for the delay. However, through day 2, recovery of the Δ*tatA* mutant from the lungs decreased. For the remainder of the study, a slight increase in CFU was detected in the lungs. However, at least one mouse out of the five tested for each time point starting on day 2 had no *Y. pestis* recovered from the lungs (Fig. 9A). A similar delay was observed for trafficking of the mutant to spleens (Fig. 9B). On day 4, one mouse did show a high bacterial burden. However, the majority of bacteria were recovered from spleens between days 5–9. As was the case observed for the lungs, at least one mouse for each time point (except for day 7), Δ*tatA* bacteria were not recovered from the spleens (Fig. 9B).

### Screening the *Y. pestis* genome for Tat motifs

To determine how the deletion of the *tatA* gene could affect capsule formation or other known virulence factors, the CO92 *Y. pestis* genome was screened for proteins containing the Tat motif (SRRXFLK) [7]. A previous study employing the TATFIND computer program identified 19 *Y. pestis* proteins predicted to be secreted via the Tat translocation pathway [58]. None of the proteins identified in this study as Tat secreted proteins appears to be directly linked to defect in capsule, biofilm formation, or other known *Y. pestis* virulence factors. Additionally, there is sufficient evidence to suggest that there is some degree of fluidity in the composition and organization of this domain [59]–[62]. Therefore, we decided to conduct an *in silico* search allowing more flexibility at each position. Table 5 lists nine proteins which contain Tat-like motifs in the N terminal region, four that are unique to this study. From our search, we identified several proteins that have been associated with the Tat translocation system in other bacteria (TauA, SufI, DmaA and NapA). From the newly identified Tat secreted candidates; we did not establish any direct links to the phenotypes observed for the *Y. pestis* mutant. However, there were several hypothetical proteins identified that warrant further characterization, such as YPO2150, a LysR regulator.

**Table 5:** *Y. pestis* proteins containing putative Tat motifs.

Motif*	Protein	Product
SRRSFLQ	TauA	taurine transporter substrate binding subunit**
SRRSFLQ	SufI	repressor protein for FtsI**
TRRKFLM	YPO0986	hypothetical protein**
SRRLALL	YPO2150	LysR family transcriptional regulator
SRREFIQ	DmsA	putative dimethyl sulfoxide reductase chain A protein**
SRRDFMK	NapA	nitrate reductase catalytic subunit**
TRRDALA	YadG	putative ABC transporter ATP-binding protein
SRRLAIL	YPO3648	putative 2-hydroxy-3-oxopropionate reductase
TRRIFIL	YPO0009	putative membrane transport protein

*Underlined letters indicate substitution with a similar amino acid from the consensus Tat motif (SRRXFLK).

**Indicates a protein that overlaps with a previous study [58] that identified predicated Tat substrates from the CO92 *Y. pestis* genome.

## Discussion

In this study, we examined the role of the Tat translocation system in a fully virulent *Y. pestis* strain. The disruption of the Tat pathway by the in-frame deletion of the *tatA* gene led to several interesting phenotypes. The morphology of the mutant bacteria was severely altered and tended to have a more bacillus shape than the typical shape of *Y. pestis* (Fig. 2). The effect this structural alteration would have specifically on virulence of the Δ*tatA* strain remains to be determined.

The change in shape of the Δ*tatA* mutant is likely due to the defect in cell division as was previously described for *tat* mutations in *E. coli, Burkholderia thailandensis*, and *Helicobacter pylori* [25], [46], [47]. In screening the *Y. pestis* genome for Tat motifs, the Suf1 protein sequence contained a putative Tat signal [58]. Suf1 was previously shown to be transported by the Tat system [60] and to also suppress cell division [63]. However, *E. coli* mutants in the *suf1* gene do not show these cell morphology defects [46], [63].

In addition to the altered morphology of the bacteria, the Δ*tatA* bacteria had a defect in biofilm integrity. The mutant strain was able to synthesize a biofilm; however, the adherence of the biofilm to the substrate was weak and could easily be dislodged (Fig. 3). Defects in biofilm formation have been observed in other bacteria when mutated in *tat* genes [17], [20], [22], [64], [65]. Currently, the exact reason for the loss of biofilm adherence in the *Y. pestis* Δ*tatA* mutant remains to be determined. For *tat* mutants in other bacteria, the decrease in biofilm production was due to loss of flagella motility or pili-twitching [20], [22]. However, *Y. pestis* is non-motile due to a mutation within the *flhD* gene [45].

The distribution and/or localization of the F1 antigen of the Δ*tatA* mutant was also affected despite the Caf1 or other related proteins not possessing an obvious Tat motif. The F1 antigen is synthesized on the bacterial surface through a chaperone/usher pathway [48], [66]. When performing Western analyses with an anti-F1 antibody and proteins of lysed whole cell or supernatants, a band corresponding to the F1 protein was still observed with the Δ*tatA* mutant, but the intensity was less than with the parenteral strain (Fig. 4A). In addition, when examining these strains by IFM or IEM with an antibody to the F1 protein, both the wild-type and mutant strains were highly labeled. However, the amount of membrane associated F1 antigen detected with the Δ*tatA* mutant was diminished (Fig. 5).

It appears that the F1 antigen protein was able to be secreted but unable to localize properly to the outer membrane surface of *Y. pestis*. The protein would then diffuse away from the cell. It has been previously reported that the Caf1A usher is necessary for assembly of F1 antigen onto the surface of the bacteria but not for secretion into the extracellular media [48], [67]. Additionally, various novel chaperone/usher loci have been described for *Y. pestis* that play some role with biofilm and F1 capsule synthesis [68]. Perhaps the initial assembly and anchoring of the F1 protein onto the surface of the *Y. pestis* Δ*tatA* mutant is altered due to loss of some chaperone/usher transported by the Tat system.

When examining the *Y. pestis* Δ*tatA* for virulence defects, the mutant was severely attenuated in a bubonic model of infection. The LD_50_ for the Δ*tatA* mutant was 10^7^ times higher as compared to the parental strain (Table 3). We demonstrated that this defect was due specifically to the loss of the *tatA* gene through restoring virulence via complementation. When the Δ*tatA* mutant was tested in pneumonic models of plague, contrasting results between delivery methods were observed. When whole-body small-particle aerosol challenges were performed, the LD_50_ for the Δ*tatA* mutant would be greater than 14 times of the wild-type CO92 strain (Table 3). We were not able to calculate a reliable LD_50_ measurement from the survival curve for challenged mice due to a lack of a monotonic response. However, other attenuated strains of *Y. pestis* have demonstrated a poor dose response in animal models of plague challenge [31], [69], [70]. Though the mutant was not as attenuated via aerosol challenge as was observed with the bubonic model, this is not unprecedented. Differences in virulence have been observed frequently with other *Y. pestis* mutants dependent upon the routes of challenge [31], [43], [55], [71]–[73].

In contrast to the differences in LD_50_ measurements observed with the other models of challenge, the LD_50_ for the Δ*tatA* mutant was only 1.7 times higher than the wild-type strain by intranasal challenge (Table 3). However, the mutant did display attenuation by other measurements of virulence following intranasal instillation: TTD (Table 4) and delayed dissemination and recovery from organs following challenge (Fig. 9). Interestingly, the Δ*tatA* mutant virulence data are very similar to the results previously reported with a Δ*pla* mutant in regards to minimal difference between LD_50_ values but large differences in TTD and CFU recoveries following intranasal challenge [74].

The intranasal challenge model is useful to study pathogenesis of infectious diseases, including pneumonic plague [74]–[78]. It is possible that the presence of bacteria, both wild-type and Δ*tatA* mutant, within the inner ear may be due to the delivery method. For wild-type *Y. pestis*, pneumonic plague and death occurs so rapidly that disease from the inner ear is not able to progress to meningitis. In contrast, infection with the Δ*tatA* mutant is slowed which allows the bacteria to persist within the inner ear and then eventually reach the brain. Interestingly, a recent article described the ability of *Y. pestis* to create a localized anti-inflammatory state that creates a protective environment for itself and other non-pathogenic bacteria in the immediate vicinity [79]. Perhaps the Δ*tatA* mutant, though attenuated by other methods of challenge, becomes an “opportunistic pathogen” when able to gain access for a prolonged time to the inner ear of a mouse and create a suitable environment for it to infect the host.

Challenges by intranasal instillation versus small-particle aerosol differ in many aspects. Namely, the sizes of the particles generated differ between the two routes and would therefore colonize different parts of the lung and body. The smaller particles generated by a whole-body aerosol chamber would be inhaled deeper into the lung. In contrast, the particles from intranasal challenges would by much larger and would infect both the upper and lower respiratory tract and be much more likely to be swallowed and infect the digestive system. Similar to the results from our current study, mice that were challenged by intranasal delivery with *Burkholderia pseudomallei* also showed a high incidence of meningitis [80]. In addition, other studies have shown differences between intranasal or aerosol delivery in dissemination of bacterial pathogens [80], [81] or inflammation response to allergens [82] in mice.

It would be tempting to speculate that the loss of virulence associated with the Δ*tatA* mutant is due to the altered F1 antigen. However, it has been shown by others that loss of the *Y. pestis* F1 antigen had little to or no difference in virulence in mice [72], [83]–[87]. In contrast, Sebbane et al. demonstrated that *Y. pestis* deleted of the *caf1M1A1* operon are affected in their ability to cause bubonic plague in mice following flea bites [88]. As the Δ*tatA* mutant was severely hindered following subcutaneous infection and displays an altered F1 antigen, it would be interesting to determine if it would be similarly defective via a flea bite route of infection. In addition, a recent study demonstrated a *caf1* deletion mutant in the CO92 strain of *Y. pestis* was attenuated by bubonic and pneumonic (intranasal) models of infection depending on the strain of mouse [77]. Our current challenge studies with the Δ*tatA* mutant were limited to Swiss Webster mice. It would be of interest to extend these studies to additional mouse strains as described by Weening at al. [77] to determine if the Δ*tatA* mutant would be more attenuated by the intranasal challenge model of plague.

Based upon proteomic and bioinformatic analysis of *E. coli* and *B. subtilis*, as much as 5–8% of secreted proteins are translocated by the Tat pathway [89]. A previous study using the TATFIND computer program, identified a total of 19 putative Tat secreted proteins [58]; however, none of the candidates seemed likely to be effectors of the phenotypes observed in the mutant. To identify *Y. pestis* Tat secreted proteins that may be responsible for the loss of virulence, biofilm adherence, or the altered F1 protein, the *Y. pestis* CO92 genome was screened for proteins containing the Tat-like motifs within the N-terminal portion substituting similar amino acids for the consensus motif sequence. Nine proteins were identified during this screen five of which have homologs demonstrated to be Tat translocation products in other bacterial species [19], [63], [90], [91]. However, many of the identified *Y. pestis* proteins from both of these studies are hypothetical or potential putative proteins of broad function. For instance, YPO2150 is a predicted transcriptional regulator and a member of the LysR family which can regulate capsule, fimbriae, and biofilm production in other bacterial pathogens [92]–[94]. In addition, novel chaperone/usher loci were recently described for *Y. pestis* [68]. Perhaps the Tat pathway interacts with these proteins and is able to directly or indirectly affect virulence.

Obviously, other Tat translocated proteins must exist in *Y. pestis* which affect pathogenesis. Recently, other proteins which utilize the Tat system but lack the signal motif were identified [59]. A more thorough examination utilizing more sophisticated methods would need to be pursued to identify *Y. pestis* Tat secreted proteins. It is likely that some of these proteins may be important virulence factors. Finally, this study further demonstrates the possibility of targeting the Tat pathway for novel therapeutics not only for *Y. pestis* but for many other bacterial pathogens.
